# Learning curve patterns generated by a training method for laparoscopic small bowel anastomosis

**DOI:** 10.1186/s41077-016-0017-y

**Published:** 2016-05-25

**Authors:** Jose Carlos Manuel-Palazuelos, María Riaño-Molleda, José Luis Ruiz-Gómez, Jose Ignacio Martín-Parra, Carlos Redondo-Figuero, José María Maestre

**Affiliations:** Hospital Virtual Valdecilla, Avda. de Valdecilla s/n, 39008 Santander, Spain

**Keywords:** Learning curve, Laparoscopic surgical procedure, Anastomosis surgical, Simulation procedural, Assessment educational, Instructional design

## Abstract

**Background:**

The identification of developmental curve patterns generated by a simulation-based educational method and the variables that can accelerate the learning process will result in cost-effective training. This study describes the learning curves of a simulation-based instructional design (ID) that uses ex vivo animal models to teach laparoscopic latero-lateral small bowel anastomosis.

**Methods:**

Twenty general surgery residents were evaluated on their performance of laparoscopic latero-lateral jejuno-jejunal anastomoses (JJA) and gastro-jejunal anastomoses (GJA), using swine small bowel and stomach on an endotrainer. The ID included the following steps: (1) provision of references and videos demonstrating the surgical technique, (2) creation of an engaging context for learning, (3) critical review of the literature and video on the procedures, (4) demonstration of the critical steps, (5) hands-on practice, (6) in-action instructor’s feedback, (7) quality assessment, (8) debriefing at the end of the session, and (9) deliberate and repetitive practice. Time was recorded from the beginning to the completion of the procedure, along with the presence or absence of anastomotic leaks.

**Results:**

The participants needed to perform 23.8 ± 6.96 GJA (12–35) and 24.2 ± 6.96 JJA (9–43) to attain proficiency. The starting point of the learning curve was higher for the GJA than for the JJA, although the slope and plateau were parallel. Further, four types of learning curves were identified: (1) exponential, (2) rapid, (3) slow, and (4) no tendency. The type of pattern could be predicted after procedure number 8.

**Conclusions:**

These findings may help to identify the learning curve of a trainee early in the developmental process, estimate the number of sessions required to reach a performance goal, determine a trainee’s readiness to practice the procedure on patients, and identify the subjects who lack the innate technical abilities. It may help motivated individuals to become reflective and self-regulated learners. Moreover, the standardization of the ID may help to measure the effectiveness of learning strategies and make comparisons with other educational strategies.

## Background

During the past decade, many studies have strengthened the evidence supporting the use of simulation-based training in surgery [1, 2]. Research has shown improvement in learning clinical, procedural, and behavioral skills [3]; transfer of learning from the simulation lab to clinical settings [4, 5]; the potential to improve quality of care [6]; a decrease in intra- and postoperative complications; and reduced length of hospital stay [7] when compared with standard instructional methods.

In response to these results, the American College of Surgeons (ACS) and the Association of Program Directors in Surgery (APDS) in the United States of America (USA) have made efforts to enhance resident training by implementing the National Surgical Skills Curriculum in the USA. This curriculum has been carefully structured and designed by content experts to enhance resident training through a web-based open platform and reproducible simulations [8]. As a standardized ready-to-use skills training program, it represents a milestone in surgical education that has the potential to reduce costs of development [9].

Despite the benefits of the National Surgical Skills Curriculum, the overall rate of its adoption in surgical residency programs is still lagging behind. Many institutes have incorporated simulation labs, but training opportunities are still limited due to the lack of faculty protected time, significant costs, and resident work-hour restrictions [10]. Consequently, research is shifting from evaluating the effectiveness of simulation to finding the most efficient methodologies for training surgical teams. In this regard, and to address these challenges, the Consortium of the ACS Accredited Education Institutes (AEI) and the Committee on Simulation of the Association for Surgical Education (ASE) were launched. The AEI Consortium, which was founded in the USA, is a global network of 77 institutes. The initial institutes were accredited by the ACS in June 2006 to promote patient safety, develop new education and technologies, identify best practices, and promote research and collaboration. The Committee on Simulation of the ASE, which was established in the USA in 2009, represents over 190 medical schools and institutes throughout Canada and the USA. Both share the mission of advancing simulation-based surgical education internationally, and one of their goals is to foster innovative research projects and educational programs that promote the identification of best practices [11]. In addition, they offer cost-effective solutions to address challenges associated with the widespread adoption of surgical simulations [12].

Until now, studies have not focused on implementation strategies or ways to improve the efficiency of simulation-based training. Nevertheless, evidence-based instructional design (ID) has become increasingly important when aiming for specific learning outcomes over a specific period of time [13]. In surgical training, learning curves are used to plot the number of attempts required to master a procedure, and a better understanding of the learning curve is essential to demonstrate surgical training [14]. Many difficult surgical procedures have a “flattened” learning curve, and trainees may undergo a training period without achieving the “plateau” of proficiency [15]. Furthermore, the learning rate for laparoscopic skills may vary with the training method and might slow down after a certain number of repetitions. In this regard, experienced surgeons did not improve their error or the economy of movement scores on a virtual reality training system after several repetitions, indicating the absence of progression for these parameters [16]. The clinical importance of this concept is that patients should not be exposed to surgeons operating during the early phase of their training. It is critical that learning curves are not used to justify the need for “hands-on” experience or to rationalize complications under the false notion that they are acceptable while acquiring new skills [16]. The challenge is to employ teaching strategies and provide enough time to ensure the proficiency or expertise has been achieved in the simulation lab before operating on patients [14]. Therefore, once an ID is developed, it should be tested to ensure that it meets the learning objectives and is effective when used with participants. We believe that the identification of the specific developmental curves generated by different training methods is the first step to design evidence-based, cost-effective educational programs.

This study describes the types of developmental curves generated by a defined simulation-based ID using ex vivo animal models to teach laparoscopic latero-lateral small bowel anastomosis. Small bowel anastomoses were selected because they are one of the most common procedures performed in gastric and intestinal, elective, and emergency general surgery. Laparoscopy has gained wide acceptance because it allows a faster return to normal activity and diet, reduced hospital stay, and reduced rate of complications (e.g., hernia, adhesive small bowel obstruction, postoperative pain, emesis) when compared to open access [17]. Nevertheless, even though the procedure is performed using a meticulous technique, anastomosis leakage is a common complication that results in significant morbidity and mortality (22 % mortality rate among patients with leakage vs. 7.2 % mortality rate among those without leakage) [18]. We hypothesized that trainees would display different learning curves.

## Methods

### Setting and context

The research was conducted at an ACS/AEI at a university hospital in Spain. The hospital offers resident physician training as well as two fellowship programs in surgery, 900 beds and more than 50 clinical specialties and is affiliated with medical and nursing schools [19]. After 6 years of medical training, candidates have to complete a 5-year general surgery residency program (the first year of residency introduces specialist training in surgery). Residency training programs in Spain are coordinated by the National Surgery Commission following requirements of the National Council of Specialties, the advisory body in health sciences for the Ministry of Health and Education. Every hospital must develop its own program following the Ministry’s directives [20].

### Trainees and residency program

Twenty general surgery residents (women, 60 %; men, 40 %) participated in the study. The resident program at our institution includes 11 sequential modules that follow the National Surgery Commission Curriculum and the ACS/APDS Surgery Resident Skills Curriculum. Each module is conducted during a specific period of the residency program and integrates knowledge acquisition and simulation lab practice with clinical practice. The study took place during the “Basic Laparoscopic Intestinal Anastomoses Module,” which includes small bowel and gastric laparoscopic anastomosis. This module is conducted during the second and third years of residency and comprises six sessions (4 h each) of individual practice in the simulation lab each year (48 h). The module is scheduled in two consecutive sessions during the first, second, and fourth trimesters. Practice is mandatory and takes place in the afternoon, once clinical work is completed. The rationale behind this program is that daily practice for several consecutive days results in the completion of a standardized exercise while minimizing fatigue for novices [21], and subsequent, repetitive deliberate practice and robust feedback promotes mastery at learning [22]. In our hospital, residents do not apply the specific competencies acquired during a module on patients until the theoretical and simulation-based sections of the module have been completed. Under this arrangement, residents do not practice small bowel laparoscopic anastomoses on patients until the end of their third year. During the study, they practiced laparoscopic appendectomy and cholecystectomy, as well as small bowel and gastric anastomoses through laparotomy (and other open abdominal surgery techniques learned in the previous modules), which may have had confounding effects on the rate of learning. This variable was the same for all subjects in the study.

### Procedures

Residents were evaluated on their performance of laparoscopic latero-lateral jejuno-jejunal anastomosis (JJA) and gastro-jejunal anastomosis (GJA). All anastomoses were 5 cm long. They were performed using ex vivo swine small bowel and stomach on an endotrainer. All samples had a standard length of 12 cm. Continuous absorbable (3/0) suture was performed.

In a previous prospective study, the time required to perform an anastomosis with no loose sutures, edge eversion, or leakage in relation to training time was almost parallel for both procedures [23].

### Instructional design

The ID was based on Kolb’s model of experiential learning [24], a revision of Bloom’s taxonomy of educational objectives [25], Ericsson’s deliberate and repetitive practice theory [26], and Rudolph et al.’s theories on establishing a safe container for learning in simulation [27], and conducting feedback and debriefing [28]. The training course was designed according to measurable objectives and clinical standards of care [29] and included these theories and concepts based on the framework to teach procedural skills developed by the Center for Medical Simulation (CMS) to promote consistent structure, process, and educational outcomes [30]. Thus, it comprised the following sequential steps:

1. Provision of a bibliography and prerecorded videos demonstrating the surgical technique to facilitate knowledge acquisition of the targeted objective (first level of Bloom’s taxonomy for knowledge-based goals). The materials were sent 15 days in advance for the residents to study and watch on their own. The journal articles and book chapters described and demonstrated actual clinical standards in small bowel anastomoses (to restore intestinal continuity and prevent anastomotic leaks).
2. Introduction to the simulation session (also known as the pre-simulation briefing) in order to establish and maintain an engaging context for learning (CMS approach). The specific practices that were identified as useful included clarifying objectives, the simulation environment (i.e., simulators, equipment, location of supplies, etc.), roles, confidentiality, and expectations. This involved establishing a “fiction contract” (in which the instructor committed to make the situation as real as possible, while acknowledging its limitations, and the participant committed to act as if everything was real), attending to logistic details, expressing respect for the learners, and showing interest in their perspective.
3. Discussion of the articles and a review of the procedure using the video to facilitate the comprehension of the information previously provided (second level of Bloom’s taxonomy for knowledge-based goals).
4. Live demonstration of the critical steps of the surgical technique by the instructor to facilitate procedural knowledge (knowledge of subject-specific techniques and methods of the revised Bloom’s taxonomy; this drew attention to the fundamental role of imitation in skill acquisition).
5. Hands-on practice to advance the ability to physically manipulate the laparoscopic instruments and skillfully perform a quick, accurate, and highly coordinated surgical technique (Bloom’s taxonomy of educational objectives for skills-based goals).
6. In-action structured feedback (within practice encounters) to promote the learning of a complex skill in the early stages including trial and error (Bloom’s psychomotor domain).
7. Time measurement and quality assessment of the anastomosis following the clinical standards described below.
8. Debriefing at the end of each training session to reflect on the experience and conceptualize what went well and what should be done differently next time (second and third stages of Kolb’s theory). The debriefing followed a “good judgment” approach, which combined feedback with genuine inquiry. It specified a rigorous reflection process that would help trainees identify and resolve pressing clinical and behavioral dilemmas raised by the simulation (CMS concepts and theories).
9. Deliberate and repetitive practice with the specific objectives detected during the previous session to facilitate expert performance (Ericsson’s theory).

### Assessment

Times were recorded from the beginning to the completion of the procedure, and the presence or absence of anastomotic leaks was noted. Samples were filled with colored water to test the tightness of the anastomoses. A previous study established the benchmark for performance standards in the lab following extensive training with abdominal simulators to master the technique. It was defined by the mean anastomotic time for end-to-end anastomoses (50 min) in animals that survived for 2 weeks. At the end of that period, a reoperation was undertaken to verify whether there were any leaks, obstructions, or adhesions [23, 31]. The residents were considered to have reached proficiency when an anastomosis was completed within the upper limit of the benchmark confidence interval (CI 60.5 min) without leakage.

The most important technical factor that can influence anastomotic healing and the prevention of leakage is the accurate union of the two bowel ends. This is best achieved by meticulous technique that includes accurate seromuscular apposition, size and spacing between each suture, and tension between the ends [18]. These factors were used to guide feedback, but the data were not recorded for this study.

### Statistical analysis

Means, median, standard deviation (SD), and interquartile range were employed for numerical variables. In addition, the Wilson Score was used to determine CIs and percentages for qualitative variables, while the Mann-Whitney *U* test was utilized to compare medians. The learning curve of each trainee was determined by using the time of the procedures along with polynomial regression and was adjusted to the fifth degree [32]. To obtain a graphical representation of the four learning curves, locally weighted scatterplot smoothing (LOWESS) was used. LOWESS utilizes multiple regression models that enable smoothing of the data. Each point along the regression curve was produced by a weighted linear least-squares regression to fit the data in the neighborhood of the local point, with the weights declining as *x* values moved farther from the *x* value of the local point [33]. R program (R Foundation for Statistical Computing, Vienna, Austria) was used for all statistical analyses [34]; we applied package car for nonparametric regression [33]. *p* < 0.05 was considered statistically significant.

### Ethical approval and informed consent

The study protocol (code: 2014.216) was approved by the Ethics and Research Committee of the Autonomous Region of Cantabria (Spain), and informed consent was obtained from all participants.

## Results

### Overall results

A total number of 476 GJA procedures and 483 JJA procedures were performed. Each participant completed an average of 48 ± 15.3 anastomoses, including the cases performed after reaching the standards. The number of procedures was slightly lower among male residents (42 ± 14.6) compared to female residents (52 ± 15.0), although the difference was not significant (*p* = 0.176 with the Mann-Whitney *U* test). The average training time was 73.7 min (SD 32.99, range 24–255) for GJA and 57.4 min (SD 20.43, range 17–155) for JJA.

### GJA

The participants needed to perform 23.8 ± 6.96 procedures (range = 12–35) to attain proficiency. Eighty-five percent of participants attained the standards after 30 anastomoses. Eight residents required between 12 and 20 repetitions, nine residents between 21 and 30 repetitions, and three residents between 31 and 35 repetitions.

**Fig. 1:**
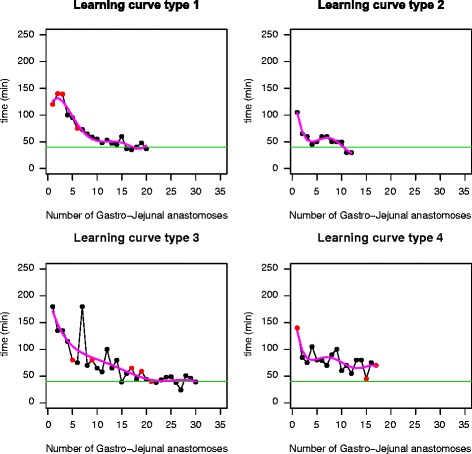
Learning curve patterns for gastro-jejunal anastomosis. *Colored red dots* indicate anastomotic leakage

An individual analysis of the data resulted in the description of four types of learning curves (Table 1): type 1: exponential (30 % of participants, CI 95 % 14.5–51.9 %); type 2: rapid (20 %, CI 95 % 8.1–41.6 %); type 3: slow (30 %, CI 95 % 14.5–51.9 %); and type 4: no tendency (20 %, CI 95 % 8.1–41.6 %). The starting point (time required to perform initial procedures) was higher among type 1 learners compared to type 2 learners, but both reached proficiency after 10 to 15 cases. Consequently, the type 1 slope (rate of learning) was steeper. Type 3 learners had a starting point similar to that of type 2 learners, but the rate of learning was slower, as these learners needed 20 to 25 cases to attain proficiency. The last group showed no clear tendency in the rate of learning, and performance did not improve consistently during the time of the study. These patterns could be predicted after procedure number 8 (Fig. 1).

**Table 1:** Learning curve patterns for laparoscopic latero-lateral gastro-jejunal (GJA) and jejuno-jejunal anastomosis (JJA)

		Learning curve 1: exponential	Learning curve 2: rapid	Learning curve 3: slow	Learning curve 4: no tendency
		Starting point: high	Starting point: low	Starting point: low	Starting point: No tendency
		Slope: steep	Slope: medium	Slope: gentle	Slope: no tendency
Time to complete first procedure (min ± SD)	GJA	145.8 ± 16.3	104.0 ± 7.4	173.3 ± 37.5	107.8 ± 35.0
	JJA	135.0 ± 15.0	62.7 ± 30.9	85.3 ± 26.3	75.0 ± 26.0
Time to complete final procedure (min ± SD)	GJA	46.5 ± 8.9	44.2 ± 15.3	47.0 ± 6.0	71.2 ± 20.1
	JJA	47.0 ± 14.7	42.0 ± 1 8.5	50.7 ± 14.5	61.8 ± 10.8
Number of residents	GJA	6	4	6	4
	JJA	3	9	3	5

*SD* standard deviation

### JJA

The participants needed to perform 24.2 ± 6.96 procedures (range = 9–43) to achieve proficiency. Eighty-five percent of participants attained standards after 34 anastomoses. Nine residents required between 9 and 20 repetitions, four residents required between 21 and 30 repetitions, and seven residents required between 31 and 43 repetitions.

**Fig. 2:**
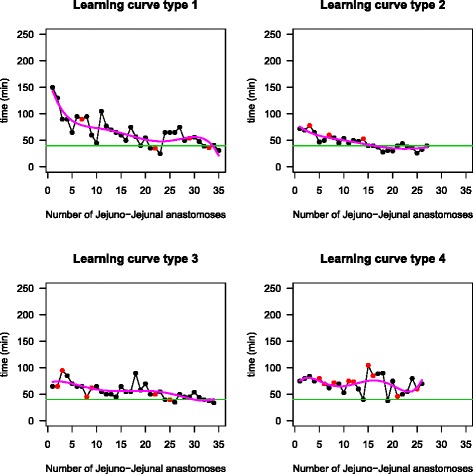
Learning curve patterns for jejuno-jejunal anastomosis. *Colored red dots* indicate anastomotic leakage

The individual analysis also enabled identification of the same four types of learning curves (Fig. 2): type 1: exponential (15 % of participants, CI 95 % 5.2–36.0 %); type 2: rapid (45 % of participants, CI 95 % 25.8–65.8 %); type 3: slow (15 % of participants, CI 95 % 5.2–36.0 %); and type 4: no tendency (25 % of participants, CI 95 %: 11.2–46.9 %). The differences in start time and the number of cases needed to achieve proficiency are shown in Table 1. As seen for GJA, the patterns of JJA could be predicted after procedure number 8.

### GJA and JJA combined

**Fig. 3:**
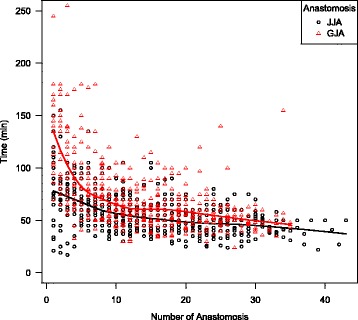
Comparison of the overall learning rate of latero-lateral gastro-jejunal anastomosis and jejuno-jejunal anastomosis

Figure 3 combines the overall learning curves of both procedures. The starting point for GJA was higher, although the slope and plateau were parallel.

## Discussion

The characteristics of the ID of a simulation-based training course may influence the performance level that the participants can achieve as well as the time needed to attain that level [29]. Our results showed that residents had to perform an average of 23.8 procedures to attain proficiency in GJA and 24.2 for JJA, while 85 % of them met the standards after 30 GJA and 34 JJA. An individual analysis of anastomotic time enabled description of four types of learning curves (exponential, rapid, slow, and no tendency) that could be predicted after procedure number 8.

In another study that used a standardized technique to teach laparoscopic bowel anastomoses, the learning process required 40 procedures with a simulator [31], instead of the 23.8 and 24.2 in our study. We think the difference may lie in the fact that the study aimed to standardize the suturing technique used for the anastomosis and not to define a comprehensive ID. In a series registered in our simulation lab between 2004 and 2007, the average training time to complete GJA and JJA was 87.2 and 72.7 min, respectively [23]. The reduction of training time found in the present study (73.7 min for GJA and 57.4 min for JJA) followed the redesign and standardization of the ID using the educational concepts and theories described above. To correlate the ID with learning outcomes, it is important to define the methodology used [13]. A critical issue when defining the ID is to clearly differentiate the tools used for learning from the actual educational methods [35]. In surgical simulations, this has been a challenge, as the simulator itself has frequently been regarded as the educational method. On the other hand, the same simulators (e.g., endotrainers, virtual simulators, mannequins) can be used in widely different educational methods. Therefore, the ID represents the specific techniques used for learning.

In our study, time was the principal criterion for determining the learning curve. In a systematic review of minimally invasive abdominal surgery, the procedural time was also the most commonly used variable (86 %). Other outcomes frequently measured included intraoperative outcomes (56 %), postoperative outcomes (54 %), intraoperative technical skills (17 %), and patient-oriented outcomes (49, 8 %). In our study, leakage, an intra- and postoperative outcome, was the main variable assessed, as observed in the majority of the articles revised [36]. The overall results show a typical learning curve as described in the Dreyfus Model of Skill Acquisition, with an initial phase of rapid qualification followed by another phase of slow development [37].

Despite the fact that different surgeons are likely to learn at different rates, most studies compare mean duration of the operation between groups [36]. In our study, however, four types of learning curves were identified for both types of anastomosis. The trainees with an exponential pattern (type 1) showed a higher starting point than those showing a rapid curve (type 2), but both reached the standards after a comparable number of procedures. The different starting point reveals that each person has individual experiences and backgrounds outside and inside the operating room that can lead to a different initial level of expertise. Some participants may have played video games before the study, and video game users seem to learn endoscopic techniques more quickly [38]. On the other hand, participants practiced laparoscopic appendectomy and cholecystectomy during the study, and the differences in previous experience may have influenced the starting point. Interestingly, the slope of the curve (how fast a person learns a new task) was similar for both groups, which might demonstrate that the generic skills in laparoscopy (i.e., innate psychomotor abilities) were similar among the participants in the two groups. The trainees with a slow learning curve (type 3) did not need longer time to complete the initial procedures, which might also be correlated with previous experience. However, the rate of learning was slower than that of the participants in the type 1 and 2 groups, which probably indicates lower innate psychomotor abilities. Interestingly, this group was also capable of obtaining proficiency with deliberate and repetitive practice with feedback. The type 4 trainees showed no clear tendency. This correlates with previous observations that indicate certain individuals cannot attain proficiency despite extensive training. This is a controversial issue. Do these subjects lack the abilities to develop laparoscopic technical skills, or are the ID and time allotment inadequate? This question poses a challenge for the professional bodies responsible for training and certification. If a type 4 (or 3) learning pattern is identified, instructors can use the technical factors described in the Methods section to guide formative assessment, identify common errors, and prescribe repetitive and deliberate practice until performance improves. When type 1 and 2 participants attain proficiency, they either continue to practice to reach expert or master level, operate on patients with supervision, or learn another procedure.

Figures 1 and 2 provide examples of each type of learning pattern. The percentage of individuals with a type 2 learning curve was higher for JJA (45 vs. 20 %). This result, coupled with the lesser time required to complete an anastomosis during the entire learning curve, confirms a faster rate of learning with this procedure.

These findings have potential implications for designing training programs for residents or for experienced surgeons aiming to learn new procedures.

This study has implications for how simulation training is implemented in educational curricula. To date, many simulation-based educational programs have been designed to include as many competencies as possible within the time frame available for training, without taking into consideration learning outcomes [39]. This approach may not ensure that all trainees reach proficiency (or mastery) for each competency. Knowing the average training time needed with a specific ID will help estimate the number of sessions required to reach the performance goal. Using this approach may limit the number of procedures taught using simulation to those that are more prevalent, complex, and associated with a higher risk of patient morbidity and mortality.

Another implication of this study is that it suggests a more effective use of resources available for training. The deep understanding of the different learning patterns generated by a specific ID enables early identification of individual learning needs. This can be detected early in the developmental process (after eight procedures in our results). This helps to plan the probable number of procedures required by a particular individual. Trainees who reach the desired level early can move on to another module, and trainees who need more practice may seek specific advice and feedback early on [40].

Another finding of this performance-oriented individual approach is the need to identify reliable, objective, long-term outcomes, and also develop valid and reliable tools to assess performance [41]. This is especially important for institutions that wish to (1) certify surgeons based on objective, valid, and transparent criteria and (2) implement a skills-assessment curriculum to identify individuals that may not attain proficiency despite extensive training (type 4). In the latter case, we believe assessment should be done before trainees formally enroll in a residency, preferably in medical school, to help the subjects who lack these innate abilities choose an alternative professional field [42].

The precise definition of *proficiency* in terms of learners’ achievement will determine a trainee’s readiness to proceed with patients. This also may impact patient safety, as it sets a definable milestone for transitioning to clinical practice.

Finally, creating opportunities for individualized instructor-guided training and reflection may help motivated individuals to become reflective and self-regulated learners. They can potentially have the tools to improve their own and other team members’ performance throughout their career [43].

There are several limitations of this study. The learning curve patterns resulted from an individual analysis of the participants and not from integration of all results into groups. This was due to a limited number of trainees, and additional cases are needed to validate the patterns we identified. The 2-year duration of the study was based on the timing of the actual resident training program. Future research should be planned during a shorter time period to prevent the influence of confounding effects while practicing other minimally invasive techniques. Participant characteristics such as previous experience with laparoscopic techniques, video game use, concurrent surgical activity during the study, or innate psychomotor ability testing were not evaluated and might have correlated to the different learning curves. Finally, this study evaluated one ID—there was no comparison group—and the results were compared to other ID studies described in the literature.

These findings suggest several paths for future research. Once learning curve patterns have been identified and correlated with a specific ID, studies can search for evidence of the most effective strategies within a design. Findings can be related to the sequence and elements of the ID used to build the training activity or to the educational methods within each element so that they can better support the learning needs of trainees while accelerating learning.

We propose several strategies to analyze the impact on the rate of learning. One is “part-task” training, wherein tasks are deconstructed into parts to be learned separately before practicing the procedure as a whole [44]. Another is cognitive task analysis, which identifies the knowledge, thought processes, and goal structures experts rely on during task performance [45]. Another strategy is guided experiential learning, wherein learners receive strong, early guidance through a script and storyboarded video demonstrations, procedural checklists, practice with increasingly difficult problems, and evaluations [46]. There are numerous debriefing methods that can be compared (good judgment [28], video-assisted [47], in-simulation [48], technical and cognitive [49], within-team [50], scripted [51], and a blended approach [52]). In our study, we used an ex vivo animal model, but live tissue, cadavers, or virtual simulators can also be compared [53, 54]. Other strategies can include activation and assessment of prior knowledge [55], spatial analysis and video gaming skills [56], and early detection, classification, and correction of consequential errors [57].

## Conclusions

In our series, an average of 24 procedures were needed to attain proficiency in latero-lateral JJA and GJA according to measurable objectives, clinical standards of care, and a standardized ID. However, individually, the number of cases completed did not correlate well with time and errors. In fact, trainees were able to learn laparoscopic skills at different rates. In this regard, four types of learning curves were identified that could be predicted after eight procedures. These findings suggest that determining progression from a novice level to master level for laparoscopic small bowel anastomosis does not mainly depend on the number of cases, but rather the training needs have to be individualized. While our results indicate several ID principles for developing effective simulation-based experiences, there is still a promising area for research to better understand what drives consistent learning outcomes.

## Abbreviations

ACS: American College of Surgeons
AEI: Accredited Education Institutes
APDS: Association of Program Directors in Surgery
ASE: Association for Surgical Education
CI: confidence interval
CMS: Center for Medical Simulation
GJA: gastro-jejunal anastomoses
ID: instructional design
JJA: jejuno-jejunal anastomoses
SD: standard deviation

## References

[1] Vanderbilt AA, Grover AC, Pastis NJ, Feldman M, Granados DD, Murithi LK (2014). Randomized controlled trials: a systematic review of laparoscopic surgery and simulation-based training. Glob J Health Sci.

[2] Zendejas B, Brydges R, Hamstra SJ, Cook DA (2013). State of the evidence on simulation-based training for laparoscopic surgery: a systematic review. Ann Surg.

[3] Cook DA, Brydges R, Hamstra SJ, Zendejas B, Szostek JH, Wang AT (2012). Comparative effectiveness of technology-enhanced simulation versus other instructional methods: a systematic review and meta-analysis. Simul Healthc.

[4] Sturm LP, Windsor JA, Cosman PH, Cregan P, Hewett PJ, Maddern GJ (2008). A systematic review of skills transfer after surgical simulation training. Ann Surg.

[5] Fraser K, Wright B, Girard L, Tworek J, Paget M, Welikovich L (2011). Simulation training improves diagnostic performance on a real patient with similar clinical findings. Chest.

[6] Wayne DB, Didwania A, Feinglass J, Fudala MJ, Barsuk JH, McGaghie WC (2008). Simulation-based education improves quality of care during cardiac arrest team responses at an academic teaching hospital: a case-control study. Chest.

[7] Zendejas B, Cook DA, Bingener J, Huebner M, Dunn WF, Sarr MG (2011). Simulation based mastery learning improves patient outcomes in laparoscopic inguinal hernia repair. Ann Surg.

[8] Scott DJ, Dunnington GL (2008). The new ACS/APDS skills curriculum: moving the learning curve out of the operating room. J Gastrointest Surg.

[9] Rooney D, Pugh C, Auyang E, Hungness E, Darosa D (2010). Administrative considerations when implementing ACS/APDS skills curriculum. Surgery.

[10] Korndorffer JR, Arora S, Sevdalis N, Paige J, McClusky DA, Stefanidis D (2013). The American College of Surgeons/Association of Program Directors in Surgery National Skills Curriculum: adoption rate, challenges and strategies for effective implementation into surgical residency programs. Surgery.

[11] Sachdeva AK (2010). Efforts to advance simulation-based surgical education through the American College of Surgeons—accredited education institutes. Surgery.

[12] Stefanidis D, Sevdalis N, Paige J, Zevin B, Aggarwal R, Grantcharov T (2014). Simulation in surgery: what’s needed next?. Ann Surg.

[13] Cook DA, Hamstra SJ, Brydges R, Zendejas B, Szostek JH, Wang AT (2013). Comparative effectiveness of instructional design features in simulation-based education: systematic review and meta-analysis. Med Teach.

[14] Hopper AN, Jamison MH, Lewis WG (2007). Learning curves in surgical practice. Postgrad Med J.

[15] Guillonneau BD (2005). The learning curve as a measure of experience. Nat Clin Pract Urol.

[16] Grantcharov TP, Bardram L, Funch-Jensen P, Rosenberg J (2003). Learning curves and impact of previous operative experience on performance on a virtual reality simulator to test laparoscopic surgical skills. Am J Surg.

[17] Raymond TM, Dastur JK, Khot UP, Parker MC (2008). Hospital stay and return to full activity following laparoscopic colorectal surgery. JSLS.

[18] Goulder F (2012). Bowel anastomoses: the theory, the practice and the evidence base. World J Gastrointest Surg.

[19] Martin-Parra JI, Manuel-Palazuelos JC, Maestre JM, Gómez-Fleitas M, Del Moral I (2013). Changing the paradigm in health care education: Hospital Virtual Valdecilla. J Surg Educ.

[20] Digestivo CnNdCaGyA. ORDEN SCO/1260/2007, de 13 de abril, por la que se aprueba y publica el programa formativo de la especialidad de Cirugía General y del Aparato Digestivo. BOE.110:19864-73.

[21] Kang SG, Ryu BJ, Yang KS, Ko YH, Cho S, Kang SH (2015). An effective repetitive training schedule to achieve skill proficiency using a novel robotic virtual reality simulator. J Surg Educ.

[22] Eppich WJ, Hunt EA, Duval-Arnould JM, Siddall VJ, Cheng A (2015). Structuring feedback and debriefing to achieve mastery learning goals. Acad Med.

[23] Rodriguez-Sanjuan JC, Manuel-Palazuelos JC, Fernandez-Diez MJ, Gutierrez-Cabezas JM, Alonso-Martin J, Redondo-Figuero C (2010). Assessment of resident training in laparoscopic surgery based on a digestive system anastomosis model in the laboratory. Cir Esp.

[24] Armstrong E, Parsa-Parsi R (2005). How can physicians’ learning styles drive educational planning?. Acad Med.

[25] Anderson LW, Krathwohl D (2001). A taxonomy for learning, teaching and assessing: a revision of Bloom’s taxonomy of educational objectives.

[26] Ericsson KA (2008). Deliberate practice and acquisition of expert performance: a general overview. Acad Emerg Med.

[27] Rudolph JW, Raemer DB, Simon R (2014). Establishing a safe container for learning in simulation: the role of the presimulation briefing. Simul Healthc.

[28] Maestre JM, Rudolph JW (2015). Theories and styles of debriefing: the good judgment method as a tool for formative assessment in healthcare. Rev Esp Cardiol.

[29] Lioce L, Meakim CH, Fey MK, Chmil JV, Mariani B, Alinier G (2015). Standards of best practice: simulation standard IX: simulation design. Clin Sim Nur.

[30] Center for Medical Simulation (2015). Simulation instructor training.

[31] Hamad MA, Mentges B, Buess G (2003). Laparoscopic sutured anastomosis of the bowel. Surg Endosc.

[32] Weisberg S (2014). Applied linear regression.

[33] Fox J, Weisberg HS (2011). An R companion to applied regression.

[34] The R project for statistical computing (2015) http://www.r-project.org. Accessed 17 Jan 2016.

[35] Chiniara G, Cole G, Brisbin K, Huffman D, Cragg B, Lamacchia M (2013). Simulation in healthcare: a taxonomy and a conceptual framework for instructional design and media selection. Med Teach.

[36] Harrysson IJ, Cook J, Sirimanna P, Feldman LS, Darzi A, Aggarwal R (2014). Systematic review of learning curves for minimally invasive abdominal surgery: a review of the methodology of data collection, depiction of outcomes, and statistical analysis. Ann Surg.

[37] Dreyfus SE (2004). The five-stage model of adult skill acquisition. B Sci Technol Soc.

[38] Lynch J, Aughwane P, Hammond TM (2010). Video games and surgical ability: a literature review. J Surg Educ.

[39] Sancho R, Rabago JL, Maestre JM, Del Moral I, Carceller JM (2010). Integración de la simulación clínica en el programa formativo de la especialidad de Anestesiología y Reanimación. Rev Esp Anestesiol Reanim.

[40] Anderson JM, Aylor ME, Leonard DT (2008). Instructional design dogma: creating planned learning experiences in simulation. J Crit Care.

[41] Vassiliou MC, Feldman LS, Andrew CG, Bergman S, Leffondré K, Stanbridge D (2005). A global assessment tool for evaluation of intraoperative laparoscopic skills. Am J Surg.

[42] Grantcharov TP, Funch-Jensen P (2009). Can everyone achieve proficiency with the laparoscopic technique? Learning curve patterns in technical skills acquisition. Am J Surg.

[43] Ericsson KA (2015). Acquisition and maintenance of medical expertise: a perspective from the expert-performance approach with deliberate practice. Acad Med.

[44] Johnson KB, Syroid ND, Drews FA, Ogden LL, Strayer DL, Pace NL (2008). Part task and variable priority training in first-year anesthesia resident education: a combined didactic and simulation-based approach to improve management of adverse airway and respiratory events. Anesthesiology.

[45] Tjiam IM, Schout BM, Hendrikx AJ, Scherpbier AJ, Witjes JA, van Merrienboer JJ (2012). Designing simulator-based training: an approach integrating cognitive task analysis and four-component instructional design. Med Teach.

[46] Craft C, Feldon DF, Brown EA (2014). Instructional design affects the efficacy of simulation-based training in central venous catheterization. Am J Surg.

[47] Hamad GG, Brown MT, Clavijo-Alvarez JA (2007). Postoperative video debriefing reduces technical errors in laparoscopic surgery. Am J Surg.

[48] Van Heukelom JN, Begaz T, Treat R (2010). Comparison of postsimulation debriefing versus in-simulation debriefing in medical simulation. Simul Healthc.

[49] Bond WF, Deitrick LM, Eberhardt M, Barr GC, Kane BG, Worrilow CC (2006). Cognitive versus technical debriefing after simulation training. Acad Emerg Med.

[50] Boet S, Bould MD, Sharma B, Revees S, Naik VN, Triby E (2013). Within-team debriefing versus instructor-led debriefing for simulation-based education: a randomized controlled trial. Ann Surg.

[51] Cheng A, Hunt EA, Donoghue A, Nelson-McMillan K, Nishisaki A, Leflore J (2013). Examining pediatric resuscitation education using simulation and scripted debriefing: a multicenter randomized trial. JAMA Pediatr.

[52] Eppich W, Cheng A (2015). Promoting excellence and reflective learning in simulation (PEARLS): development and rationale for a blended approach to health care simulation debriefing. Simul Healthc.

[53] Hall AB (2011). Randomized objective comparison of live tissue training versus simulators for emergency procedures. Am Surg.

[54] Stefanidis D, Yonce TC, Green JM, Coker AP (2013). Cadavers versus pigs: which are better for procedural training of surgery residents outside the OR?. Surgery.

[55] Hailikari T, Katajavuori N, Lindblom-Ylanne S (2008). The relevance of prior knowledge in learning and instructional design. Am J Pharm Educ.

[56] Millard HA, Millard RP, Constable PD, Freeman LJ (2014). Relationships among video gaming proficiency and spatial orientation, laparoscopic, and traditional surgical skills of third-year veterinary students. J Am Vet Med Assoc.

[57] Tang B, Hanna GB, Cuschieri A (2005). Analysis of errors enacted by surgical trainees during skills training courses. Surgery.

